# The Significant Impact of Biomass Burning Emitted Particles on Typical Haze Pollution in Changsha, China

**DOI:** 10.3390/toxics13080691

**Published:** 2025-08-20

**Authors:** Qu Xiao, Hui Guo, Jie Tan, Zaihua Wang, Yuzhu Xie, Honghong Jin, Mengrong Yang, Xinning Wang, Chunlei Cheng, Bo Huang, Mei Li

**Affiliations:** 1 Hunan Province Ecology and Environment Monitoring Center, Ministry of Ecology and Environment Key Laboratory of Heavy Metal Pollution Monitoring, Changsha 410014, China; 13787198525@163.com (Q.X.); 18807312113@163.com (J.T.); yuzhu032@sina.com (Y.X.); jingyingying888@163.com (H.J.); yangmengrong@126.com (M.Y.); 2 School of Energy Conservation and Safety, Guangdong Polytechnic of Environmental Protection Engineering, Guangzhou 510655, China; zaihuawang@163.com; 3 College of Environment and Climate, Institute of Mass Spectrometry and Atmospheric Environment, Guangdong Provincial Engineering Research Center for Online Source Apportionment System of Air Pollution, Jinan University, Guangzhou 510632, China; wxn410@126.com (X.W.); limei2007@163.com (M.L.); 4 Guangzhou Hexin Analytical Instrument Company Limited, Guangzhou 510530, China; huangbo0717@hotmail.com

**Keywords:** biomass burning, haze, single particles, source apportionment

## Abstract

In this study, typical haze pollution influenced by biomass burning (BB) activities in Changsha in the autumn of 2024 was investigated through the mixing state and evolution process of BB particles via the real-time measurement of single-particle aerosol mass spectrometry (SPAMS). From the clean period to the haze period, the PM_2.5_ concentration increased from 25 μg·m^−3^ at 12:00 to 273 μg·m^−3^ at 21:00 on 12 October, and the proportion of total BB single particles in the total detected particles increased from 17.2% to 54%. This indicates that the rapid increase in PM_2.5_ concentration was accompanied by a concurrent increase in the contribution of particles originating from BB sources. The detected BB particles were classified into two types based on their mixing states and temporal variations: BB1 and BB2, which accounted for 71.7% and 28.3% of the total BB particles, respectively. The analysis of backward trajectories and fire spots suggested that BB1 particles originated from straw burning emissions at northern Changsha, while BB2 particles were primarily related to local nighttime cooking emissions in Changsha. In addition, a special type of K-containing single particles without K cluster ions was found closely associated with BB1 type particles, which were designated as secondarily processed BB particles (BB-sec). The BB-sec particles contained abundant sulfate and ammonium signals and showed lagged appearance after the peak of BB1-type particles, which was possibly due to the aging and formation of ammonium sulfate on the freshly emitted particles. In all, this study provides insights into understanding the substantial impact of BB sources on regional air quality during the crop harvest season and the appropriate disposal of crop straw, including conversion into high-efficiency fuel through secondary processing or clean energy via biological fermentation, which is of great significance for the mitigation of local haze pollution.

## 1. Introduction

Biomass burning (BB) particles are a major component of atmospheric fine particulate matter (PM_2.5_) and represent the largest global source of carbonaceous aerosols [1,2]. BB emissions can significantly influence ambient air quality and radiative forcing [3,4], emitting large quantities of volatile organic compounds, black carbon, brown carbon, and various toxic species such as carcinogenic polycyclic aromatic hydrocarbons (PAHs) and water-soluble cyanides. These pollutants can enter aquatic systems through diffusion or wet and dry deposition [5], posing significant risks to ecosystems [6]. Therefore, many field studies have focused on BB events and evaluated their impact on haze formation and atmospheric chemistry.

In the Pearl River Delta, China, BB particles contribute approximately 7–11% of organic aerosol (OA) concentrations, with higher levels observed at suburban monitoring sites than in urban centers [7]. In Beijing, BB contributes 50–70% of OA in suburban areas and 30–40% in urban areas, leading to severe pollution events under unfavorable meteorological conditions [8,9]. Due to the cyclical nature of agricultural practices, BB in China exhibits strong seasonal characteristics. Crop residue burning during harvest seasons is a key contributor to regional haze. For instance, a study in the Guanzhong Plain found that BB organic aerosol accounted for 31.9% of OA in autumn and 15.3% in winter [10]. Similarly, the prevalence of BB particles increases during the summer wheat harvest in northern China [11].

Traditional offline analysis methods for monitoring BB particles suffer from low temporal resolution and cannot provide real-time source apportionment or information on particle mixing states. Aerosol mass spectrometry (AMS) offers quantitative chemical composition data. When combined with receptor models such as Positive Matrix Factorization, AMS enables source apportionment from online datasets. OA components resolved via AMS typically include hydrocarbon-like OA, oxygenated OA, biomass burning OA, and cooking-related OA. BBOA has been reported to contribute between 10% and 60% of OA in PM_1_ and often dominates during pollution episodes [8,12,13]. Single-particle aerosol mass spectrometry (SPAMS) provides another online measurement of BB single particles, which can provide the detailed mixing states and chemical compositions of single particles, and can be used to identify BB particles through characteristic mass spectral peaks and to quantify their number concentrations. In addition, SPAMS can ionize refractory components (such as BC and metals) and resolve their mixing states with other components. Generally, BB particles exhibit distinct spectral features, including strong potassium ion signals (^39^K^+^) and marker cluster ions such as ^26^CN^−^, ^42^CNO^−^, ^45^CH_3_O_2_^−^, ^59^C_2_H_3_O_2_^−^, ^71^C_3_H_3_O_2_^−^, as well as ^113/115^K_2_Cl^+^ and ^213/215^K_3_SO_4_^+^ [14,15]. These signatures enable source differentiation and in situ, number-based source apportionment. Existing SPAMS studies in China have reported that BB particles account for 11% to 60% of total particle number concentrations, with substantial variation across regions and seasons [15,16,17,18]. While reported studies have explored the contribution of BB emissions to PM_2.5_ concentration and composition, significant knowledge gaps still exist regarding the evolution of BB particles' mixing states along their transport pathways. Specifically, it remains unclear how freshly emitted BB particles are transported to monitoring sites after being released during intense events causing rapid PM_2.5_ increases and how their mixing states evolve during atmospheric aging processes. Furthermore, distinguishing the contributions from different BB source types remains challenging. Specifically, the attribution of particles to regional agricultural residue burning versus local residential cooking activities is unresolved, and the variation in the relative proportions of these distinct BB particle types during pollution episodes also needs to be characterized.

Hunan Province is one of China’s major agricultural regions, and the total grain cultivation area reached 11.79 million acres in 2024, with rice as the dominant crop, grown primarily in a double-cropping system (early and late rice). The early-season rice covered 2.99 million acres, yielding 7.33 × 10^9^ kg, the highest in the country. To investigate the characteristics of BB particles and evaluate their impact on atmospheric quality in Hunan, we conducted monitoring during the autumn of 2024 (September to November) at a site in Changsha, using a single-particle aerosol mass spectrometer. BB particles were identified from ambient aerosol samples, and their characteristic species, classification, and temporal concentration trends were analyzed to provide scientific support for future environmental policy and emission control strategies.

## 2. Sampling and Data Analysis

### 2.1. Sampling and Measurements

**Figure 1:**
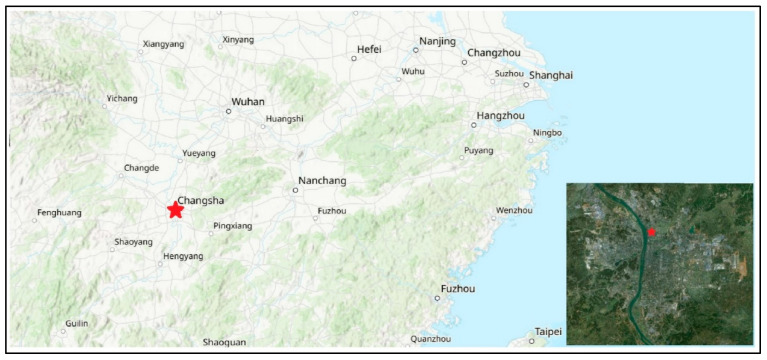
The location of sampling site in Changsha, China.

An online single-particle aerosol mass spectrometer (SPAMS) (Guangzhou Hexin Instrument Co., Ltd., Guangzhou, China) was deployed on the rooftop of the Kaifu District Government Office Building in Changsha (28.26304° N, 112.9919° E) (Figure 1). The monitoring campaign was conducted from 01:00 on 11 September to 24:00 on 22 November 2024. The site, located in the northern part of Changsha’s Kaifu District, is co-located with the national ambient air quality monitoring station at Wujialing. It is located at the east of the Xiangjiang River and is primarily surrounded by residential and commercial zones, intersected by two major roadways, making it representative of the urban atmospheric environment.

Ambient air was drawn through a PM_2.5_ cyclone inlet and delivered via a 1 cm diameter conductive silicone tube into the SPAMS system. The instrument operated continuously, providing 24 h online measurements of individual particle composition, aerodynamic diameter, and number concentration. To ensure data quality, the instrument was calibrated and tested prior to deployment. Routine inspection and maintenance were performed throughout the campaign, including daily checks of inlet pressure, laser energy, and mass spectral stability. The inlet pressure was typically maintained slightly above 2.0 Torr; when it dropped below 1.80 Torr, the orifice plate was manually cleaned or replaced. Mass drift beyond instrument specifications was corrected through mass calibration. Laser energy was manually adjusted to remain within the target range of 500 μJ ± 10% whenever necessary.

Auxiliary data supporting the SPAMS analysis included conventional air quality parameters (PM_2.5_, PM_10_, O_3_, NO_2_, SO_2_, and CO) as well as meteorological data (wind direction, wind speed, temperature, and atmospheric pressure), all obtained from the co-located national monitoring site. Biomass burning-related fire hotspot data were acquired from NASA’s Fire Information for Resource Management System (FIRMS) (https://firms.modaps.eosdis.nasa.gov, accessed on 9 June 2025). Air mass backward trajectory simulations were performed using the Hybrid Single-Particle Lagrangian Integrated Trajectory (HYSPLIT) model developed by NOAA (https://www.ready.noaa.gov/HYSPLIT.php, accessed on 9 June 2025), using GDAS meteorological data with the following model parameters: a total run time of 24 h and an arrival height of 300 m.

### 2.2. SPAMS Measurement and Clustering Algorithm

The SPAMS (type: 0515) employed in this study was manufactured by Guangzhou Hexin Instrument Co., Ltd. A brief introduction of its principle is provided here [19]. Atmospheric particles are introduced into the instrument through a 0.1 mm diameter orifice under vacuum conditions. The particles first pass through an aerodynamic lens, which imparts a velocity that correlates with their aerodynamic diameter. After exiting the lens system, the particles pass sequentially through two continuous-wave laser beams (532 nm), generating two scattering signals. The time interval between these signals, combined with the known 6 cm spacing between the laser beams, enables the calculation of particle velocity. A standard velocity-to-diameter calibration curve is used to determine the aerodynamic diameter. After sizing, particles enter the mass spectrometry chamber, where a pulsed UV laser (266 nm) is triggered at the appropriate time to ionize the particles. The resulting positive and negative ions are detected by a bipolar time-of-flight mass spectrometer, yielding a comprehensive single-particle analysis. Each particle record includes detection time, aerodynamic diameter, scattering signal intensity, and both positive and negative mass spectra.

Based on prior studies of the characteristic composition of biomass burning (BB) particles, two criteria were employed for the preliminary identification of BB particles in this study: (1) the presence of a mass spectral peak at *m*/*z* = 39 (K^+^) and (2) the presence of at least one peak at *m*/*z* = −26 (CN^−^) or −42 (CNO^−^). These criteria significantly narrowed the pool of candidate BB particles, thereby reducing computational workload. The selected particles were then subjected to cluster analysis. For each resulting subclass, mass spectra, particle size distributions, and temporal trends in number concentration were examined to exclude non-BB particle types.

The clustering method used in this study was based on an improved version of the ART-2a algorithm [20]. In the traditional ART-2a algorithm, cluster centers are initialized by randomly selecting spectra from the dataset, which can introduce bias and hinder convergence. In this study, the improved algorithm defines initial cluster centers using the “mutual nearest neighbor” principle: If two spectra are each other’s closest match within the dataset, the midpoint between them is designated as the initial cluster center. This approach identifies the most stable spectral pairings, enhancing intra-cluster coherence and inter-cluster differentiation. Although more computationally intensive in theory, the algorithm takes full advantage of matrix computation capabilities, resulting in significantly improved clustering accuracy and efficiency over the original ART-2a method.

### 2.3. The Identification of Biomass Burning Particles

This study employed both the characteristic ions reported from previous studies by SPAMS and the mass spectra of fresh BB particles obtained from controlled laboratory experiments in this work. Three representative crop residues, including rice straw, wheat straw, and corn stalks, were selected and naturally dried, and then approximately 20 g of each type was weighed for testing. Combustion was carried out under ambient air conditions in a custom-built combustion furnace. The resulting smoke was drawn into a custom-fabricated stainless-steel chamber (volume: 4.5 m^3^) maintained under a slight negative pressure (10^1^–10^2^ Pa). The SPAMS was connected to an external sampling port on the chamber to conduct real-time single-particle chemical analysis. A total of 159,000 BB particles were detected, and their mass spectra were commonly associated with BB marker ions, including ^39^K^+^, ^113/115^K_2_Cl^+^, ^213/215^K_3_SO_4_^+^, ^104^K_2_CN^+^, ^109/110^KCl_2_^−^, ^26^CN^−^, ^42^CNO^−^, ^45^CH_3_O_2_^−^, ^59^C_2_H_3_O_2_^−^, and ^71^C_3_H_3_O_2_^−^. According to reported studies, the K^+^ salts were mainly KCl and K_2_SO_4_ [21], and the organic tracer of BB emission was levoglucosan [22], which produced the ion fragments of ^59^C_2_H_3_O_2_^−^ and ^71^C_3_H_3_O_2_^−^ via the laser ionization [15]. These ions were subsequently used as reference markers in the identification of ambient BB particles.

## 3. Results and Discussions

### 3.1. Overview of Campaign

From 11 September to 22 November 2024, the average concentrations of air pollutants during the campaign were as follows: SO_2_ (4 μg·m^−3^), NO_2_ (17 μg·m^−3^), CO (0.9 mg·m^−3^), O_3_ (181 μg·m^−3^), PM_2.5_ (39.7 μg·m^−3^), and PM_10_ (62 μg·m^−3^). Among these, only ozone exceeded the Grade II limits defined by China’s National Ambient Air Quality Standards (160 μg·m^−3^). Daily assessments at the Wujialing site identified 8 polluted days, comprising 6 days of light pollution and 2 days of moderate pollution. Ozone (O_3_-8h) served as the dominant pollutant on 6 of these days, while PM_2.5_ dominated on the other 2 days. Meteorological conditions during the study period were characterized by an average temperature of 24.1 °C, relative humidity of 65.3%, a prevailing northwesterly wind direction, and an average wind speed of 1.1 m·s^−1^.

**Figure 2:**
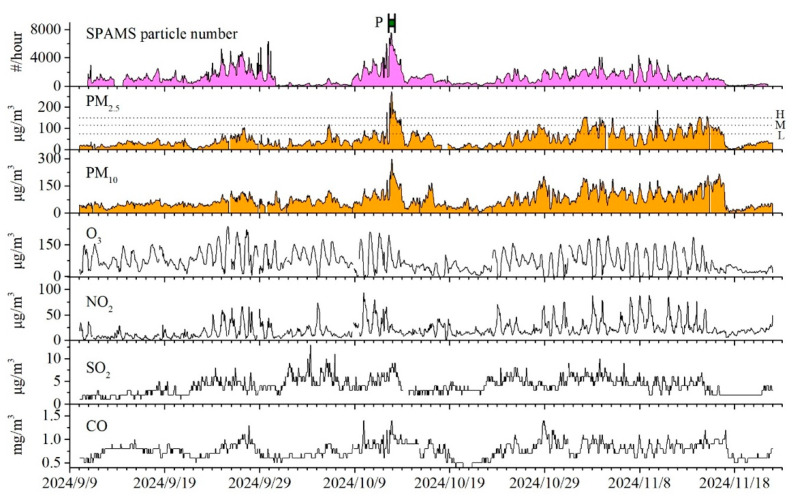
Temporal variations of atmospheric pollutants (PM_2.5_, PM_10_, O_3_, NO_2_, SO_2_, and CO) and the number count of single-particle monitored by SPAMS. # number concentration.

Temporal variation in PM_2.5_ concentrations is illustrated in Figure 2. Although the average PM_2.5_ concentration (39.7 μg·m^−3^) was below the Grade II national standard (75 μg·m^−3^), episodes of elevated concentrations were observed. Based on national classification thresholds for PM_2.5_ pollution, 75 μg·m^−3^ (light), 115 μg·m^−3^ (moderate), and 150 μg·m^−3^ (heavy), the monitoring period included a cumulative 275 h of light pollution, 66 h of moderate pollution, and 27 h of heavy pollution. The highest concentrations of PM_2.5_ were observed from 17:00 on 12 October to 07:00 on 13 October, so this period was designated as the Pollution Period in Figure 2.

**Figure 3:**
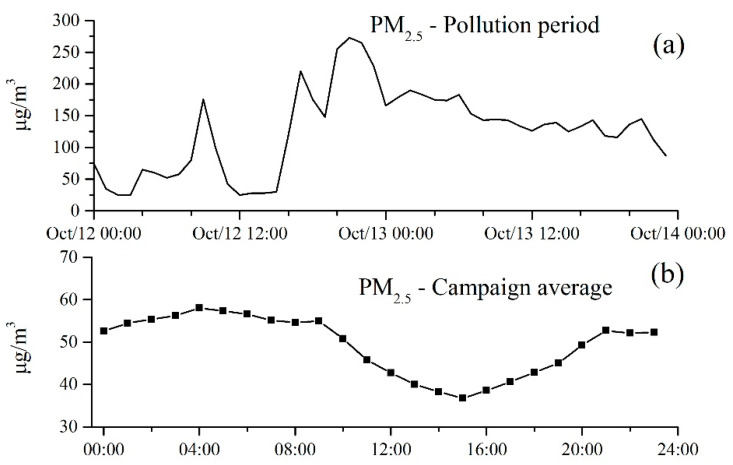
Temporal variations of PM_2.5_ concentration during haze pollution period (**a**) and the diurnal variation of PM_2.5_ concentration during the clean period (**b**).

As shown in Figure 3, PM_2.5_ concentrations exhibited a significant increase during the haze episode, surging from 25 μg·m^−3^ at 12:00 to 273 μg·m^−3^ at 21:00 on 12 October, and hourly concentrations remained above 100 μg·m^−3^ without displaying a clear diurnal pattern. In contrast, PM_2.5_ concentration in the clean period showed the diurnal cycle with high concentrations in the nighttime and low concentrations in the afternoon, reflecting stable daily sources of PM_2.5_. Such a distinct difference in PM_2.5_ in haze and clean periods strongly suggests that biomass burning acted as a potent and transient source that rapidly influenced the diurnal pattern of PM_2.5_ in the haze pollution period.

### 3.2. Mass Spectra of Biomass Burning Particles

The presence of a strong K signal is a distinguishing feature of BB particles, and this is due to the thermal transformation of the K naturally present in biomass into fine particulate-phase K salts during combustion. Under the 266 nm ionization laser employed by SPAMS, K exhibits a high ionization efficiency, resulting in prominent mass spectral peaks at *m*/*z* = +39 (K^+^) and its less abundant isotope at *m*/*z* = +41. These peaks are typically among the most intense in BB particle spectra and are widely observed across emissions from both herbaceous and woody biomass combustion sources [14,23]. In this study, a set of marker ions in both the positive and negative mass spectra was used to identify the BB-type particles. In the positive ion spectra, BB particles are observed with K-containing cluster ions, including ^113^K_2_Cl^+^, ^213^K_3_SO_4_^+^, ^94^K_2_O^+^, ^140^K_2_NO_3_^+^, and ^104^K_2_CN^+^. In the negative ion spectra, organic ions of ^26^CN^−^, ^42^CNO^−^, ^45^CH_3_O_2_^−^, ^59^C_2_H_3_O_2_^−^, ^71^C_3_H_3_O_2_^−^, and ^73^C_3_H_5_O_2_^−^ (Figure 3) are also found in fresh BB particles.

**Figure 4:**
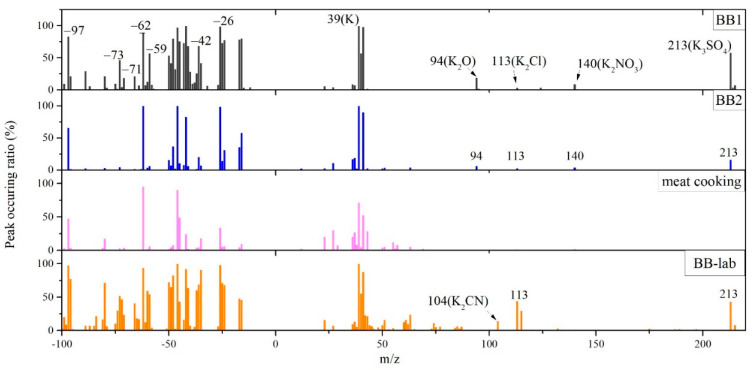
Mass spectra of fresh BB particles measured in lab, two types of BB particles from ambient measurement, and meat cooking particles identified in this study.

**Figure 5:**
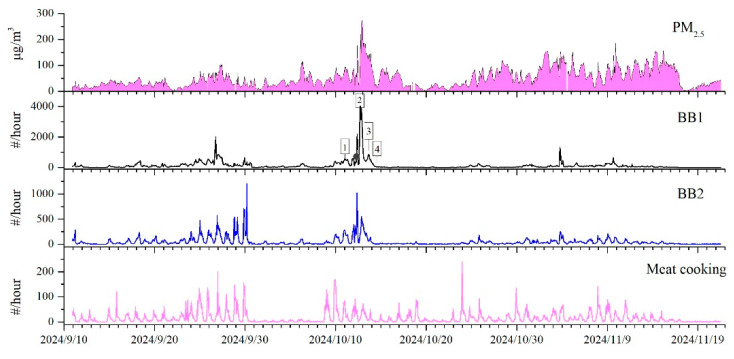
Temporal variations of PM_2.5_ mass concentration and number concentrations of BB1, BB2, and meat cooking particles. The numbers of 1–4 in the temporal variation in BB1 particles were corresponding to the four backward trajectories of 1–4 mentioned in the following Section 3.4. # number concentration.

Based on temporal variation patterns during the monitoring campaign, BB particles were classified into two distinct types: BB1 and BB2, which both contained similar K cluster ions commonly associated with BB emissions (Figure 4), while their difference lies in their negative ion spectra and temporal variations. BB1 type particles exhibited episodic (pulse-like) occurrences, and BB2 particles were characterized by periodic oscillations in number concentration (Figure 5). Furthermore, BB2 particles contained abundant organic fragment ions typically associated with biomass burning, and this was consistent with laboratory-derived BB (BB-lab) particles. However, differences were observed between BB2 and BB-lab particles. Specifically, the ^113^K_2_Cl^+^ signal in BB2 (3.8%) was substantially lower than that in BB-lab particles (44.3%), whereas ^213^K_3_SO_4_^+^ was more prevalent in BB2 (58.0%) compared to fresh BB-lab particles (43.4%). These differences suggest that BB2 particles underwent atmospheric aging, consistent with prior findings that KCl in fresh BB particles can react with acidic atmospheric species to form K_2_SO_4_ and KNO_3_ [4,24]. In addition to BB particles, meat cooking particles were also resolved, and their negative mass spectra showed strong signals of *m*/*z* 255 (C_16_H_32_O_2_^−^, palmitic acid) and *m*/*z* 281 (C_17_H_34_O_2_^−^, oleic acid), along with the abundant presence of *m*/*z* 45 (C_2_H_5_O^−^) ion. According to reported studies, these particles are attributed to emissions from meat cooking activities [25,26]. The negative mass spectra of meat cooking particles showed similar mass spectral features with BB2 particles, yet K-related cluster ions were not found in the positive mass spectra of meat cooking particles.

### 3.3. Distribution of Biomass Burning Particles

BB is a major source of atmospheric particles and an important contributor to carbonaceous aerosols globally [8,9]. Previous studies in northern China have shown that BB can contribute 46–59% to ambient organic aerosols depending on air mass trajectories. Positive matrix factorization analyses have further indicated that BB sources account for approximately 19.3% of PM_2.5_ mass concentrations [27]. In Beijing, the contribution of BB to PM_2.5_ was estimated at 15.3% during the clean period in winter, rising to 20.1% during the pollution period [28]. These studies primarily discussed mass contributions and did not address the evolution of mixing states of BB particles. Other studies have quantified the number contribution of BB particles using a particle sizer instrument. For example, BB particles were found to contribute 24% and 28% to total number concentrations in the 25–100 nm and 100–600 nm size ranges in the suburban area, respectively. In urban areas, BB contributions were lower, at approximately 13% and 20% [29].

In this study, the contributions of BB particles to total detected atmospheric particles were summarized in Table 1, which showed the hourly ratio of different types of BB particles, including BB1 (freshly emitted from straw burning), BB2 (primarily linked to cooking-related biomass combustion), and secondarily processed BB particles (BB-sec). During the entire sampling period, the average ratios of BB1 and BB2 particles in total particles were 8.2% and 3.3%, respectively, yielding a combined BB contribution of 11.5%. The maximum hourly ratio in total particles was as follows: BB1 particles reached a maximum of 64.0%, while BB2 particles peaked at approximately 24.1%. The combined BB particles reached a maximum ratio of 66.9%, which coincided with the highest mass concentration of PM_2.5_ during the pollution episode. Generally, the BB particle contribution in this study is lower than reported studies [11,15,30], and the highest ratio during the haze period in this study was similar to other SPAMS measurements [31]. The temporal trends of BB particles exhibited a pulsed pattern, characterized by short-term spikes without sustained high concentrations. From the clean period to the haze episode, the PM_2.5_ concentration increased from 25 μg·m^−3^ at 12:00 to 273 μg·m^−3^ at 21:00 on 12 October, and the proportion of total BB single particles in the total detected particles increased from 17.2% to 54%. This indicates that the rapid increase in PM_2.5_ concentration was accompanied by a concurrent increase in the contribution of particles originating from BB sources.

### 3.4. Source Analysis of Biomass Burning Particles

Open burning of crop residues accounts for approximately 20% of all biomass burning activities and constitutes a significant contributor to BB particle emissions [32]. In China, biomass burning emissions include the use of crop residues for cooking and heating, open field burning of agricultural waste during harvest seasons, and wildfires in forest or grassland areas. Emission inventory studies conducted in China suggest that straw burning and the domestic use of firewood contribute over 95% of the total BB emissions [33]. Thus, the possible sources of the two types of BB particles identified in this study were investigated by their temporal trends, mixing states, regional fire hotspot distributions, and air mass backward trajectories.

#### 3.4.1. Source Analysis of BB1 Type Particles

During the entire sampling period, a total of 212,288 BB1 particles were detected, accounting for 71.7% of all BB particles and 9.8% of the total particles detected by SPAMS. The mass spectra of BB1 particles closely resembled those of laboratory-generated straw burning particles, indicating that they most likely originated from agricultural straw combustion. As shown in Figure 5, the temporal trends of BB1 particles showed sharp and narrow peaks, implying episodic and short-term influences at the sampling site, typical of straw burning events, which are transient and non-continuous compared with stable sources such as vehicular emissions or coal combustion. The PM_2.5_ mass concentrations peaked and remained at high concentrations for 14 consecutive hours on 12 October, and this peak was consistent with the rapid increase in BB1 particle counts, with BB1 particles constituting up to 64% of all detected particles at this time, which was the highest ratio observed throughout the campaign. These findings strongly suggest that BB emissions were the primary driver of the elevated PM_2.5_ concentrations observed on 12 October.

**Figure 6:**
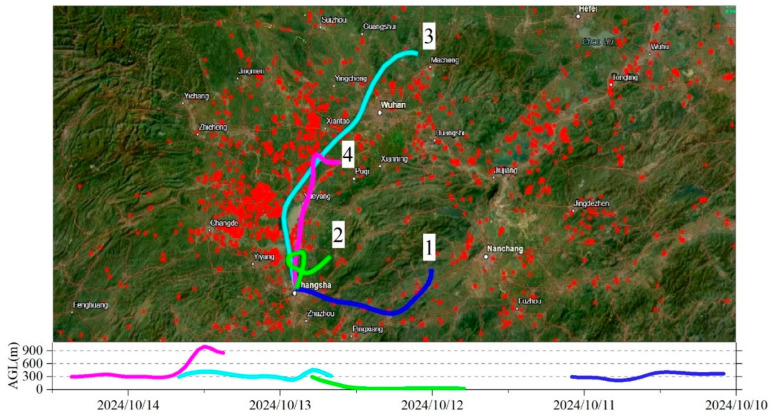
To trace the potential source of pollution event, the 24 h backward trajectories and satellite fire hotspot data were combined to illustrate the influence of BB emissions on sampling site: the air mass backward trajectories originating from 12:00 on 11 October (Blue trajectory 1), 19:00 on 12 October (Green trajectory 2), 16:00 on 13 October (Cyan trajectory 3), and 09:00 on 14 October (Magenta trajectory 4). Red dots indicate satellite-detected fire hotspots from 10 to 11 October. The bottom lines show the hourly vertical heights (above ground level, meter) of the air masses during the transportation along the four trajectories.

To trace the potential source of this pollution event, the 24 h backward trajectories and satellite fire hotspot data were combined and illustrated in Figure 6. Daily FIRMS fire maps revealed a strong connection between elevated PM_2.5_ mass concentrations and dense fire hotspot distributions, particularly over the Dongting Lake Plain, a major agricultural region in Hunan Province, where the autumn harvest typically occurs from mid-October to early November. From 9 to 11 October, extensive BB activities were observed in this area. In contrast, both hotspot density and PM_2.5_ concentrations declined after 12 October.

To further confirm the transport pathways, HYSPLIT backward trajectories were calculated at four times: 12:00 on 11 October, 19:00 on 12 October (corresponding to the BB1 peak), 16:00 on 13 October, and 09:00 on 14 October. Since FIRMS fire data are recorded in UTC (8 h behind local time), and each trajectory spans 24 h, BB activities from 10 October were also considered. The resulting air mass trajectories were combined on hotspot maps for 10–11 October (Figure 6). As shown in Figure 6, fire hotspots were densely distributed over the Dongting Lake Plain, while few hotspots were observed south of the sampling site. Before the BB1 peak, air masses (e.g., Trajectory 1) mainly originated from the south. After 17:00 on 12 October, a northward shift in wind direction occurred, as indicated by Trajectories 2 and 3. This wind shift coincided with the surge in BB1 particle concentrations and aligned with the fire hotspot distribution. Although Trajectory 4 also indicated air masses from the Dongting Lake Plain, yet, the fire hotspots had decreased by that time, so PM_2.5_ concentrations returned to normal levels. These results strongly suggest that dense BB activities in the Dongting Lake Plain were the primary source of the heavy pollution episode observed on 12 October.

#### 3.4.2. Source Analysis of BB2 Type Particles

A total of 83,798 BB2 particles were identified during the campaign, accounting for 28.3% of all BB particles and 3.9% of the total SPAMS detected particles. Compared to BB1, BB2 particles exhibited distinct spectral and temporal variations. The positive mass spectra were characterized by strong ^39^K^+^ signals and typical potassium cluster ions (e.g., ^113^K_2_Cl^+^, ^213^K_3_SO_4_^+^). The negative spectra contained abundant CN^−^ and CNO^−^ ions, while organic ions, including CH_3_O_2_^−^, C_2_H_3_O_2_^−^, C_3_H_3_O_2_^−^, and C_3_H_5_O_2_^−^) showed lower abundance than BB-lab mass spectra. The BB2 particles showed a regular diurnal pattern with higher values at night and lower concentrations during the day, similar to the temporal trend of meat cooking particles. BB2 particle count typically increased around 20:00, peaked shortly thereafter, and gradually declined by the following morning. This nighttime pattern contrasts with the irregular open field straw burning activities and suggests a different emission source. BB2 particles were likely associated with nighttime barbecue activities near the sampling site, where biomass fuels, including wood chips, rice husks, and corn cobs, were commonly used in nighttime barbecue activities.

**Figure 7:**
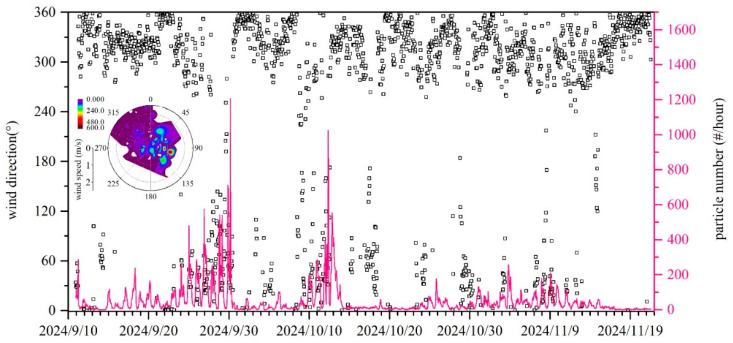
Temporal variation in BB2 particles and wind direction and the polar plot between BB2 particles, wind direction, and wind speed.

BB2 particles showed a stronger dependence on wind direction compared to BB1 particles. Figure 7 presents the connection between BB2 particles and wind direction. Elevated BB2 particles were predominantly observed during easterly and southeasterly winds, while northerly winds corresponded to lower particle count. This suggests that BB2 particles originate from local sources rather than long-range transport. Given that residential areas and downtown Changsha are located to the east and southeast of the sampling site, thus, it is reasonable to speculate that urban nighttime barbecue activities were likely the primary source of BB2 particles.

### 3.5. Aging Process of Biomass Burning Particles

**Figure 8:**
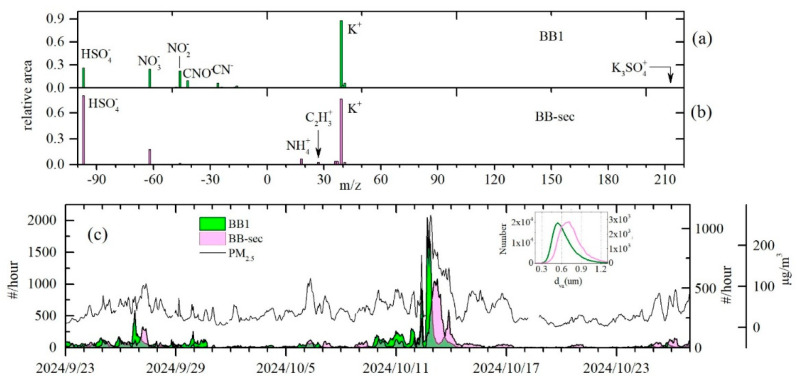
Mass spectra of BB1 type particles (**a**) and secondary BB particles (BB-sec) (**b**) and their temporal variations during entire sampling period (**c**). The inset in figure (**c**) shows the particle size distributions of BB1 and BB-sec particles. # number concentration.

In this study, the identification of BB particles relied on characteristic mass spectral signatures derived from BB-lab particles, particularly the presence of a strong ion peak of ^39^K^+^ and typical negative ions such as ^26^CN^−^ and ^42^CNO^−^. This definition is effective for the identification of primary BB particles that retain distinct emission fingerprints. However, BB particles may undergo complex physical and chemical aging processes, gradually altering their original signatures. Here, a type of K-rich particles referred to as BB-sec were studied to illustrate the aging process of fresh BB particles. As shown in Figure 8, a total of 36,193 BB-sec particles were detected. In the positive ion spectra of BB-sec particles, the ^39^K^+^ signal was prominent, and other K cluster ions typically associated with BB emissions were not observed. Instead, the strong ammonium ion signal (^18^NH_4_^+^) and minor organic ion of C_2_H_3_^+^ were found. The negative ion spectra were characterized by the strong signal of HSO_4_^−^ and a moderate NO_3_^−^ signal. The temporal trend of BB-sec particles showed consistent co-occurrence with BB1 particles, yet with lagged peak appearance. The BB-sec particles likely originated from the aging process of BB1 particles that had undergone chemical transformation via reactions with sulfate and ammonium. This was further supported by particle size distributions: BB-sec particles peak at ~690 nm, substantially larger than the ~540 nm mode of BB1, consistent with the accumulation of secondary species onto the particles. The aging process of BB1 particles might change the original BB spectral features, resulting in the formation of BB-sec particles. The formation of ammonium sulfate was possibly due to elevated ammonia emissions from BB activities. According to reported studies using thermal desorption-SPAMS systems, the heating-aged particles can decompose secondary coatings and re-expose their core emission signatures, with the proportion of BB-identified particles increasing at higher desorption temperatures [34].

In this study, the BB1 particle count initially increased as PM_2.5_ mass concentrations but declined before PM_2.5_ began to decrease during the haze pollution period on 12 October (Figure 8), and the BB-sec particle count started to increase at the same time. This suggests that a substantial fraction of BB1 particles acquired secondary coatings, altering their spectral characteristics, which resulted in this fraction of BB particles being unrecognized by the standard classification principle. Thus, the apparent decline in BB1 particles should not be interpreted as a decrease in biomass burning activity but rather as a transformation in BB particle composition and mixing state. These findings suggest the potential underestimation of BB particles after emissions due to the evolution of atmospheric processes.

Generally, BB emitted particles contain heavy metals, PAHs, elemental carbon, and nitrogen- and oxygen-containing organic compounds [35]. Numerous studies have evaluated the toxic effects and health risks associated with BB particles, investigating the toxicity distributions linked to different combustion types and biomass sources [36,37]. For instance, research isolating water-soluble and organic-soluble fractions from wood pyrolysis tar demonstrated that both fractions reduce cell viability, inducing cytotoxicity through elevated reactive oxygen species and DNA damage [38]. Additionally, the BB tracer levoglucosan has been shown to contribute significantly to particle mutagenicity, accounting for 16% of direct and 28% of indirect mutagenicity variance [39]. Toxicity equivalence assessments of PAHs emitted from different fuels indicate that BB particulate matter generally poses a higher carcinogenic risk than emissions from clean coal combustion but a lower risk than bituminous coal combustion [40]. Comparative cytotoxicity studies of PM_2.5_ from three major sources—vehicle exhaust, coal combustion, and biomass burning—reveal that while BB-derived PM_2.5_ exhibits lower toxicity than vehicle or coal-derived particles, it remains significantly more toxic than urban ambient PM_2.5_ overall, highlighting crop residue burning as a priority pollution source crucial for improving urban air quality and protecting public health [41]. High-resolution mass spectrometry studies resolving pro-inflammatory components of organic aerosols in PM_2.5_, combined with cellular assays, identify nitrogen-containing organic compounds as major inflammatory drivers, with nitroaromatic compounds playing a dominant role—compounds partly originating from biomass burning [42]. In summary, BB emissions significantly modulate PM_2.5_ toxicity, and the findings in this work demonstrate that intense BB emissions rapidly escalate PM_2.5_ concentrations, leading to haze pollution. Consequently, the health risks posed by BB particles during such episodes are likely to substantially exceed those from other emission sources. Thus, once the source pathways, transport, and atmospheric aging processes of BB particles are clearly elucidated, further research should prioritize investigating the specific health risks associated with BB particles.

## 4. Conclusions

This study investigated the characteristics of BB particles via the real-time measurement of SPAMS in Changsha during the autumn of 2024. The characteristic species, temporal variation, potential sources, and atmospheric aging process of BB particles were comprehensively discussed. During the transition from the clean period to the haze episode on 12 October, PM_2.5_ concentrations sharply increased from 25 μg·m^−3^ at 12:00 to 273 μg·m^−3^ at 21:00, concurrent with an increase in BB particles from 17.2% to 54% of total detected particles. This demonstrates that rising PM_2.5_ levels were driven substantially by BB-sourced particles. The BB particles were further classified into two subtypes based on mixing states and temporal patterns: BB1, dominant at 71.7% of total BB particles, originating from straw burning emissions in northern Changsha, and BB2, with a proportion of 28.3%, primarily linked to local nighttime cooking emissions in urban Changsha. Notably, a group of K-rich particles without other BB marker ions was identified as secondarily processed BB particles, which were characterized by abundant sulfate and ammonium signals. These BB-type particles appeared after the BB1 peak, suggesting the secondary formation of ammonium sulfate on freshly emitted BB particles. Given that crop residue burning during harvest seasons significantly contributing to PM_2.5_ loading and haze pollution, the mitigation of BB activities should be considered through the following measures: (1) Reduce open burning of rice straw during harvest periods via the regulation of local government and (2) promote centralized collection of rice straw for conversion into high-efficiency fuel through secondary processing or clean energy via biological fermentation; in addition, effective implementation requires strong governmental enforcement to mitigate haze pollution from agricultural burning.

## Figures and Tables

**Table 1:** The ratio of BB-type particles in total detected particles (%).

Type	Min	P_25_	P_50_	P_75_	Max	Mean	Std
BB1	0.0	2.1	5.6	12.0	64.0	8.2	8.3
BB2	0.0	1.4	2.5	4.4	24.1	3.3	2.7
BB1 + BB2	0.0	3.9	8.6	16.6	66.9	11.5	9.8
BB-sec	0.0	0.4	0.9	1.6	11.8	1.3	1.6
Total	0.0	5.1	10.0	18.1	68.5	12.8	10.1

## Data Availability

The observational data obtained in this study are available from the corresponding authors upon reasonable request (chengcl.vip@foxmail.com).

## Abbreviations

The following abbreviations are used in this manuscript:

BB	Biomass burning
SPAMS	Single-particle aerosol mass spectrometry
PM2.5	Atmospheric fine particulate matter
BB1	BB particles originated from straw burning emissions
BB2	BB particles related to local nighttime cooking emissions
BB-lab	BB particles derived from laboratory
BB-sec	Secondarily processed BB particles
PAHs	Polycyclic aromatic hydrocarbons
OA	Organic aerosol
AMS	Aerosol mass spectrometry
FIRMS	Resource Management System
HYSPLIT	Hybrid Single-Particle Lagrangian Integrated Trajectory
